# Feasibility of sit training for patients with severe COVID-19 pneumonia during deep sedation: A case report

**DOI:** 10.1097/MD.0000000000026240

**Published:** 2021-06-04

**Authors:** Tokio Kinoshita, Yasunori Umemoto, Yoshinori Yasuoka, Tatsuya Yoshikawa, Ken Kouda, Shinnosuke Hori, Yukio Mikami, Yukihide Nishimura, Kyohei Miyamoto, Seiya Kato, Fumihiro Tajima

**Affiliations:** a Department of Rehabilitation Medicine, Wakayama Medical University; b Division of Rehabilitation, Wakayama Medical University Hospital, 811-1 Kimiidera, Wakayama, Wakayama; c Department of Rehabilitation Medicine, Iwate Medical University, 2-1-1 Idaidouri, Yahaba-cho, Shiwa-gun, Iwate; d Department of Emergency and Critical Care Medicine, Wakayama Medical University, 811-1 Kimiidera, Wakayama, Wakayama, Japan.

**Keywords:** case report, early ambulation, rehabilitation, severe acute respiratory syndrome coronavirus 2, sitting position

## Abstract

**Rationale:**

There have been a few reports on the early rehabilitation of patients with coronavirus disease (COVID-19), and none on the effectiveness and adverse events of early mobilization for mechanical ventilation patients (other than COVID-19) during deep sedation. This report indicates that sitting without adverse events is possible in patients with severe COVID-19 pneumonia during deep sedation with muscle relaxation.

**Patient concerns:**

A 65-year-old man with a history of diabetes mellitus, lacunar infarction, and Parkinson's disease was admitted to a local hospital for pneumonia due to COVID-19. After admission, the patient was managed on a ventilator under deep sedation with muscle relaxants and sedatives. Twelve days after admission, the patient was transferred to our hospital due to his worsening respiratory status.

**Diagnosis:**

Pneumonia due to COVID-19 was diagnosed using a polymerase chain reaction–dependent method.

**Interventions:**

The day following transfer, a physical therapist started passive range of motion training and sitting.

**Outcomes:**

The period spanning his initial rehabilitation to muscle relaxant medication interruption was 9 days, and he underwent 7 rehabilitation sessions. The patient was unable to sit during only one of the 7 sessions due to pre-rehabilitation hypoxemia. In 5 of the 6 sitting sessions, PaO2/FiO2 transiently decreased but recovered by the time of subsequent blood sampling. The patient's PaCO2 decreased during all sessions. His blood pressure did not drastically decrease in any sitting session, except the first. Sputum excretion via sputum suction increased during sitting, and peak inspiratory pressure did not change.

**Lessons:**

The patient eventually died of pneumonia due to COVID-19. However, sitting during deep sedation with muscle relaxants did not cause any serious adverse events nor did it appear to cause obvious negative respiratory effects.

## 1 Introduction

In early December 2019, a patient with pneumonia from an unknown cause emerged in Wuhan, China.^[1]^ Thereafter, novel coronavirus disease (COVID-19) caused by severe acute respiratory syndrome coronavirus 2 has rapidly spread worldwide. As of December 8, 2020, 65.8 million cases and over 1.5 million deaths have been reported worldwide.^[2]^ In Japan, 163,929 total cases and 2382 deaths have been reported.^[3]^ An analysis of the Japanese COVID-19 registry (COVID-19 REGISTRY JAPAN)^[4]^ revealed that 62% of all cases were mild and did not require oxygen, 30% were moderate and required oxygen, and 9% were severe requiring intensive care with ventilation or extracorporeal membrane oxygenation, of which 7.5% were fatal.^[4]^ Patients with severe COVID-19 admitted to the intensive care unit (ICU) are at high risk of developing ICU-acquired weakness and disuse syndrome because current treatment methods include prolonged sedation for pulmonary protection, insufflation management, and neuromuscular blocking agent use.^[5,6]^ For ICU patients, it is recommended that rehabilitation be provided as early as possible.^[7]^ Some reports recommend early rehabilitation for patients with COVID-19^[6]^; however, some guidelines contraindicate rehabilitation care during deep sedation for COVID-19 patients.^[8]^ This is because dyspnea, airway clearance, skeletal muscle training, and respiratory physiotherapy aimed at promoting maintenance and recovery of activities of daily living tend to increase respiratory load. Therefore, there is no consensus on whether to recommend aggressive early rehabilitation, such as sitting on the edge of bed, for patients with COVID-19.

This report presents preliminary findings regarding the feasibility of early mobilization in a severe COVID-19 case.

## 2 Case description

A 65-year-old man with a history of diabetes mellitus, lacunar infarction, and Parkinson's disease presented to his doctor with a fever lasting 3 days. That day, he tested positive for COVID-19 and was admitted to the hospital. After admission, the patient developed respiratory failure and was placed on a ventilator under deep sedation with muscle relaxants (rocuronium) and sedatives (propofol). During ventilator management, the PaO_2_/FiO_2_ ratio (P/F) was approximately 200, indicating poor oxygenation. Twelve days after initial admission, the patient was transferred to our hospital for possible extracorporeal membrane oxygenation introduction due to his worsening respiratory status and was admitted to the ICU. The day after transfer, a physiatrist and a skilled therapist began rehabilitation therapy for expectoration and prevention of ICU-acquired weakness.

Respiratory management of patients at the beginning of rehabilitation is performed under deep sedation with muscle relaxants and mechanical ventilation (pressure-controlled ventilation, frequency 28, FiO_2_ = 0.5, inspiratory pressure above positive end-expiratory pressure (PEEP) = 15 mmH_2_O, PEEP = 13 mmH_2_O). The patient had a Richmond Agitation-Sedation Scale (RASS) of -5. Arterial blood gas values were as follows: pH = 7.416, PaO_2_ = 119 mm Hg, PaCO_2_ = 50.0 mm Hg, P/F = 238, and lactate = 1.9 mmol/L. Laboratory test results were: C-reactive protein = 9.72 mg/dL (normal range <0.3), white blood cell count = 8260/μL (normal range, 4000–9000), and hemoglobin = 11.2 g/dL (normal range, 13.0–16.6). Chest radiographs showed enhanced vascular shadows in both lung fields and mildly decreased permeability. The sequential organ failure assessment (SOFA) score was 9 (respiratory = 4, coagulation = 0, hepatic = 1, circulatory = 3, central nervous system = 1, and renal = 0) at admission. The patient was managed in the supine position during the day and in the prone position at night. In addition, the use of extracorporeal membrane oxygenation in patients was not made at the discretion of the critical care physician. Throughout early rehabilitation, approximately 2 weeks after disease onset, decreased circulating blood volume and circulatory regulatory function due to pneumonia-induced deconditioning and accumulation of alveolar secretions were suspected. Rehabilitation consisted of passive range of motion training and placement in the sitting position. The sitting position prevents ICU-acquired weakness and circulating blood volume reductions, autonomic blood pressure (BP) regulation maintenance, and promotes expectoration. Rehabilitation staff wore personal protective equipment to prevent COVID-19 transmission during the implementation of the rehabilitation program. To reduce risk of infection, the rehabilitation program was conducted in presence of minimum required staff (1 physical therapist and 1 nurse).

## 3 Assessment and intervention

Initial rehabilitation treatment was conducted under physiatrist supervision to address any adverse events that may occur during sitting. Resting BP was 91/61 mm Hg, heart rate (HR) 58 beats/min, and oxygen saturation (SpO_2_) 99%. When sitting, BP changed to 71/53 mm Hg, HR to 59 beats/min, and SpO_2_ to 97%. The patient was returned to the supine position because, while sitting, his BP decreased without an increased HR response. After BP recovery, the patient was placed in the sitting position again. Although his BP was maintained, a block associated with supraventricular extrasystoles was observed, and HR values fluctuated between 60 and 120 beats/min; therefore, the patient was returned to the supine position. After recovery, the patient was placed and held in the sitting position a third time to ensure that his BP was maintained. The total duration of sitting was 20 minutes, and SpO_2_ at the end of sitting was 96%. The overall duration (including rest) of each rehabilitation session was approximately 60 minutes. Peak inspiratory pressure at rest was 29 mmH_2_O and mean inspiratory pressure was 20 mmH_2_O, with no variation while sitting. Tidal volume at rest was approximately 370 mL and did not vary greatly while sitting (between 350 and 370 mL).

Arterial blood was collected before the start of rehabilitation, immediately before returning to the supine position (while sitting), and 15 minutes after the end of rehabilitation to assess oxygenation. P/F was 246 before rehabilitation. P/F decreased to 184 during sitting, which improved to 238 15 minutes after rehabilitation. Although the sitting position caused a transient P/F decrease, it was judged to be within the acceptable range for adverse events because the patient recovered in the supine position. The rehabilitation policy was to continue the same program until deep sedation was lifted and muscle relaxant medication was discontinued.

**Figure 1:**
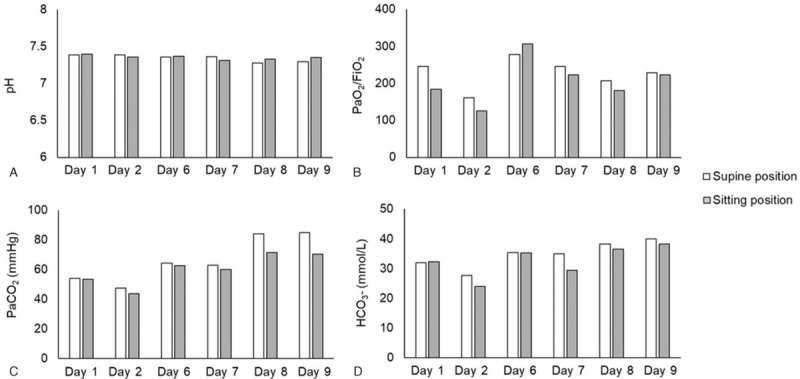
Changes in blood gas data due to placement in a sitting position. The supine position data were measured immediately prior to sitting, and the sitting position data were measured immediately before the end of each sitting session.

Nine days passed from the first rehabilitation session to muscle relaxant discontinuation, and rehabilitation sessions were performed 7 times. The patient was placed in the sitting position for > 15 minutes in all sessions but 1, due to hypoxemia (SpO_2_ 92%) before rehabilitation was performed. Blood gas data before and during sitting throughout the study are shown in Figure 1, and changes in BP are shown in Table 1. The detailed rehabilitation status for each day is shown in Supplemental Digital Content 1, and the details of the medications taken during the study period and their doses are shown in Table 2. P/F transiently decreased during sitting in 5 of 6 sessions. However, the patient recovered by the end of the day. BP decreased on days 1 and 8; however, it recovered on day 8 while maintaining the sitting position. Although tachycardia occurred during sitting session, sitting was continued because his BP remained within the acceptable range; HR recovered to the pre-sitting level after the patient was returned to the supine position. The patient's sputum excretion by sputum suction increased after sitting ended in all rehabilitation sessions except the first. Nine days after beginning rehabilitation, oxygenation mildly improved, and the patient's RASS was -4 with the interruption of muscle relaxants and respiratory management with synchronized intermittent mandatory ventilation. During rehabilitation, P/F ranged from 183 to 360.

**Table 1:** Blood pressure before and during sitting.

	Day 1	Day 2	Day 6	Day 7	Day 8	Day 9
Supine position^∗^	91/61	90/58	109/68	112/62	151/71	141/64
Sitting position^∗^^,^^†^	71/53	89/59	115/74	93/57	124/74	130/70

**Table 2:** Daily doses of medication around starting rehabilitation in intensive care unit.

Drugs	Day 0	Day 1	Day 2	Day 3	Day 4	Day 5	Day 6	Day 7	Day 8	Day 9
Propofol (mg)	3600	3600	3600	3600	3193	3114	2011	2347	2532	2880
Dexmedetomidine (μg)	480	480	480	612	759	576	484	480	480	379
Fentanyl (μg)	600	600	600	400	0	0	0	0	0	0
Morphine (mg)	0	0	0	8	24	24	24	24	24	24
Rocuronium (mg)	436	335	210	367	480	477	432	432	411	387
Norepinephrine (mg)	7.0	1.6	0	0.2	5.0	3.6	3.0	2.1	0.5	0
Remdesivir (mg)	100	100	100	100	0	0	0	0	0	0
Piperacillin/tazobactam (g)	16/2	16/2	16/2	16/2	16/2	16/2	16/2	16/2	16/2	16/2
Dexamethasone (mg)	6.6	6.6	0	0	0	0	0	0	0	0
Unfractionated heparin (Unit)	11100	13200	14400	15200	16800	17800	19200	19200	20300	14600
Levodopa (mg)	150	150	150	150	150	150	150	150	150	150

The same rehabilitation program was continued after discontinuing muscle relaxants. Nineteen days after starting rehabilitation, sedation was discontinued, but oxygenation worsened; consequently, sedation was restarted, and the patient was managed with pressure-controlled ventilation. Afterward, the rehabilitation program was limited to range of motion training because the respiratory function was not expected to improve, and the patient's respiratory condition was unstable, with oxygen saturation levels dropping below 90% even in the side-lying position. Thereafter, no improvement in oxygenation was observed, and the patient was died due to COVID-19 severe pneumonia 35 days after beginning rehabilitation.

Written informed consent was obtained from the patient's family for the publication of this case report. This study conforms to all case report guidelines, and reports required information accordingly.

## 4 Discussion

There have been a few reports on the early rehabilitation of patients with COVID-19,^[9,10]^ and none on the effectiveness and adverse events of early mobilization for mechanically ventilated patients (other than COVID-19) during deep sedation. The present study indicates that sitting without adverse events is possible in patients with severe COVID-19 pneumonia during deep sedation (RASS of -5) with muscle relaxation.

Respiratory management of moderate to severe COVID-19 patients is based on lung protection. Therefore, low tidal volume, low driving pressure, and PEEP addition are essential, as in acute respiratory distress syndrome treatment.^[11]^ In this patient, both supine therapy and respiratory management for lung protection were implemented. In COVID-19 pneumonia, spontaneous respiration increases may decrease intrathoracic pressure, resulting in pulmonary edema, which may worsen respiratory status.^[12]^ In patients with existing lung injury, regional forces generated by respiratory muscles may produce injurious effects on a regional level. In addition, an increase in transmural pulmonary vascular pressure swings caused by inspiratory effort may worsen vascular leakage.^[13]^ The patient was under deep sedation management with muscle relaxants and pressure-controlled ventilation. Therefore, the possibility of inducing spontaneous breathing or increasing inspiratory pressure associated with sitting was low. Peak inspiratory pressure was stable and remained below 30 mmH_2_O, even in the sitting position. Therefore, for COVID-19 patients, sitting under deep sedation is unlikely to produce immediate adverse effects on COVID-19 pneumonia, due to its low risk of increasing intra-alveolar pressure.

Orthostatic hypotension and arrhythmia occurred during only the first sitting session in this case. Greenleaf reported that orthostatic hypotension occurs after the fourth day of bed rest,^[14]^ and it is known that lying in bed decreases arterial baroreceptor responsiveness.^[15]^ In this case, the patient had already been forced to rest for 2 weeks before beginning sitting rehabilitation. Furthermore, the muscle relaxant rocuronium has side effects of bradycardia, sinus bradycardia, ventricular extrasystoles, and hypotension, and the sedative propofol has side effects of bradycardia, suggesting that these drugs may have affected circulatory control. In this case, BP and arrhythmia were improved when the patient was repositioned in a supine position after his BP decreased during sitting. However, due to the possibility of hypotension and arrhythmias persisting after returning patients to the supine position, it may be best to perform the initial sitting session with a physiatrist and a therapist. In this patient, hypotension did not occur after the second session. The sitting position causes blood to move to the lower extremities and reduces venous return, which triggers the baroreceptor reflex.^[16]^ Although he was sitting for only 1 day, placement in an anti-gravity position may have adjusted the patient's circulatory control.

Although retention of airway secretions is an uncommon COVID-19 symptom,^[17]^ physical therapy is indicated when COVID-19 patients have large amounts of airway secretions that they are unable to expectorate themselves. Physical therapy improves airway clearance in patients on ventilatory control and positioning changes promote the elimination of airway secretions.^[18]^ This patient exhibited excessive alveolar secretions. Emergency staff performed daytime and night-time positioning such as supine and side-lying, for alveolar recruitment purposes, while the physical therapist was responsible for sitting position placement. In this patient, the addition of the sitting position promoted expectoration and likely promoted alveolar recruitment.

After 24 hours of lying down, a 5% to 15% decrease in plasma volume has been observed, and even after 20 days, the decrease was 15%.^[14]^ It is unclear whether sitting was beneficial for preventing plasma volume loss or ICU-acquired weakness, as the patient developed pulmonary fibrosis and had a fatal outcome 35 days after rehabilitation initiation. Mortality in ICU patients was reportedly 33% with an initial SOFA score of 9, 50% with a score of 10, and 95% with a score of ≥11.^[19]^ Furthermore, older age and underlying diseases, including cardiovascular disease, diabetes mellitus, chronic respiratory disease, and hypertension, have been associated with increased mortality in COVID-19 patients.^[20]^ This patient was 65 years old, his initial SOFA score was 9, and he had underlying diabetes mellitus, which may have led to a serious outcome.

## 5 Conclusion

In this report of a patient with severe COVID-19, sitting rehabilitation during ventilator ventilation management with deep sedation and muscle relaxants did not cause any serious adverse events or obvious negative respiratory effects. In the future, it may be necessary to start sitting on the edge of the bed during deep sedation in patients with severe COVID-19. However, a large-scale study is required to confirm such outcomes associated with sitting rehabilitation.

## Acknowledgments

The authors thank the clinical nursing staff in the acute care unit for supporting the rehabilitation therapy. The authors also thank Editage (http://www.editage.com) for English language editing.

## Author contributions

**Conceptualization:** Tokio Kinoshita, Yasunori Umemoto, Yukihide Nishimura.

**Investigation:** Tokio Kinoshita, Yoshinori Yasuoka, Ken Kouda, Shinnosuke Hori, Yukio Mikami, Seiya Kato.

**Methodology:** Tatsuya Yoshikawa, Ken Kouda.

**Project administration:** Yasunori Umemoto.

**Supervision:** Kyohei Miyamoto, Fumihiro Tajima.

**Validation:** Yoshinori Yasuoka, Tatsuya Yoshikawa, Shinnosuke Hori, Yukio Mikami, Yukihide Nishimura.

**Writing – original draft:** Tokio Kinoshita, Yasunori Umemoto.

**Writing – review & editing:** Yukihide Nishimura, Kyohei Miyamoto, Seiya Kato, Fumihiro Tajima.

## Supplementary Material

Supplemental Digital Content
