# Supplementary material for: Feasibility of sit training for patients with severe COVID-19 pneumonia during deep sedation: A case report
Source: Medicine (Baltimore). 2021 Jun 4;100(22):e26240. doi: 10.1097/MD.0000000000026240 (PMC8183700; doi:10.1097/MD.0000000000026240)
Supplement: Supplemental Digital Content [file medi-100-e26240-s001.docx]

**Supplemental Digital Content 1.** Details of each day’s rehabilitation

| Day 1 | Performed passive ROM training before sitting on the edge of the bed. After the first sitting, the patient’s BP decreased, so he returned to the supine position to rest. In the second sitting position, a block appeared, so he returned to the supine position and rested. The third time, the patient had a block, but his blood pressure was stable, so he continued sitting. The patient was provided with a total of 20 minutes of sitting. |
| --- | --- |
| Day 2 | Performed passive ROM training before sitting on the edge of the bed. The patient had no significant changes in vitals during sitting on the edge of the bed, and one sitting session was performed for 20 minutes. The patient was suctioned once in the sitting position. |
| Day 3 | The patient’s SPO_2_ before rehabilitation was 92%, which was low, so sitting was not performed. Only ROM training of the extremities was performed. |
| Day 6 | Performed passive ROM training before sitting on the edge of the bed. The patient had no significant changes in vitals during sitting on the edge of the bed, and one sitting session was performed for 20 minutes. The patient was suctioned once in the sitting position. |
| Day 7 | Performed passive ROM training before sitting on the edge of the bed. The patient had no significant changes in vitals during sitting on the edge of the bed, and one sitting session was performed for 20 minutes. The patient was suctioned once in the sitting position. |
| Day 8 | Performed passive ROM training before sitting on the edge of the bed. BP 151/71 before rehabilitation; BP decreased to 124/74 in the sitting position but increased while discontinuing sitting. One sitting was performed for 20 minutes. The patient was suctioned once in the sitting position. |
| Day 9 | Performed passive ROM training before sitting on the edge of the bed. Before rehabilitation, BP, HR, and SPO_2_ were 141/64, 90, and 99%, respectively. After 10 minutes of sitting, they were 130/74, 180, 98%, respectively, and tachycardia occurred, so the patient was returned to the supine position for rest. The patient was placed in the sitting position again but became tachycardic within 5 minutes, so the sitting position was completed for the day. The patient was suctioned once in the sitting position. |

ROM, range of motion; BP, systolic arterial pressure/diastolic arterial pressure (mmHg); SpO_2_, oxygen saturation; HR, heart rate (beat/min).
